# Identification of an effective siRNA target site and functional regulatory elements, within the hepatitis B virus posttranscriptional regulatory element

**DOI:** 10.1186/1743-422X-7-216

**Published:** 2010-09-08

**Authors:** Nattanan Panjaworayan, Sunchai Payungporn, Yong Poovorawan, Chris M Brown

**Affiliations:** 1 Department of Biochemistry, Faculty of Science, Kasetsart University, Bangkok, Thailand; 2 Department of Biochemistry, Faculty of Medicine, Chulalongkorn University, Bangkok, Thailand; 3 Center of Excellence in Clinical Virology, Department of Pediatrics, Faculty of Medicine, Chulalongkorn University, Bangkok, Thailand; 4 Department of Biochemistry, University of Otago, Dunedin, New Zealand

## Abstract

**Background:**

Infection with hepatitis B virus (HBV) is major public health concern. The limitations of available antiviral drugs require development of novel approaches to inhibit HBV replication. This study was conducted to identify functional elements and new siRNA target sites within the highly conserved regions of the 533 base post-transcriptional regulatory element (PRE) of HBV RNAs.

**Results:**

Computational analysis of the PRE sequence revealed several conserved regulatory elements that are predicted to form local secondary structures some of these within known regulatory regions. A deletion analysis showed that sub-elements of the PRE have different effects on the reporter activity suggesting that the PRE contains multiple regulatory elements. Conserved siRNA targets at nucleotide position 1317-1337 and 1329-1349 were predicted. Although the siRNA at the position 1329-1349 had no effect on the expression of reporter gene, the siRNA target site at the position 1317-1337 was observed to significantly decrease expression of the reporter protein. This siRNA also specifically reduced the level of cccDNA in transiently HBV infected cells.

**Conclusion:**

The HBV PRE is likely to contain multiple regulatory elements. A conserved target within this region at 1317-1337 is an effective siRNA target.

## Introduction

Hepatitis B virus (HBV) infection is a major cause of hepatocellular carcinoma and liver cirrhosis worldwide [1]. HBV vaccination can prevent new infections, but effective antiviral drugs are required for the large number of HBV infected people. Current licensed therapies such as interferon-α, lamivudine and adefovir dipivoxil have been found to have many limitations. For example, interferon-α is found to have a limited use for a narrow range of patients and is associated with a number of adverse effects whereas a long-term use of lamivudine and adefovir dipivoxil could cause drug-resistant variants of HBV [2]. Novel approaches for inhibiting HBV replication are therefore urgently needed.

Currently, RNA interference (RNAi) has been emerged as a potential technique for developing nucleic acid-based gene silencing therapeutics for treatment of viral diseases [3-7]. RNAi is a specific mechanism for down-regulation of gene expression. It is evolutionally conserved from plants to mammals. The RNAi process is initiated by short double-stranded RNAs (dsRNAs) that lead to the sequence-specific inhibition of their homologous genes [8]. Previous studies with HBV have shown effective inhibition of HBV replication in mammalian tissue culture and in a mouse model by using synthetic small interfering RNAs (siRNAs) [9-11] and siRNA expression plasmids, which the siRNAs are generated from short hairpin RNA transcripts (shRNAs) and processed into active siRNAs by Dicer in the cytoplasm of cells [12-16].

The HBV genome contains four large overlapping open reading frames that encode for five major proteins namely core, large surface, middle surface, small surface and X proteins [17]. Other smaller proteins may be generated by splicing or as regulatory small reading frames [18]. Several sites located on different HBV transcripts were demonstrated to be siRNA target sites [15,19,20]. Mostly, these siRNA target sites were predicted along the sequence of HBV genome using bioinformatics programmes, which are based on certain characteristics of ideal siRNAs such as two nucleotides 3' overhang, low GC content (36-52%), base preference at position 3, 10, 13 and 19, but not by the target mRNA functionality [19,21,22]. This approach, although effective, is limited as it does not consider conservation in the genome. Therefore target sites, although initially effective, might be able to be mutated and the virus become resistant.

**Figure 1:**
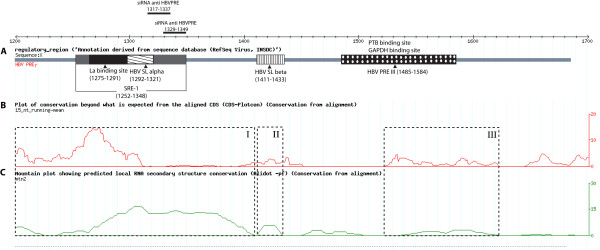
**Prediction of conserved functional elements within the HBV PRE**. (A) A schematic diagram of HBV PRE with annotation of previously reported elements: human La-binding site (nucleotides 1275-1291) [30,31], a splicing regulatory element-1 (SRE-1) (nucleotides 1252-1348), the conserved stem loop, HBV SLα (nucleotides 1292-1321) [32], the conserved stem loop HBV SLβ (nucleotides 1411-1433) [32], a subsection named PREIII (nucleotide 1485-1584) that was reported to bind to two cellular proteins *in vitro*, PTB and GAPDH [33,35]. The siRNA target sites investigated in this study were also indicated (siRNA anti HBV PRE 1317-1337 and siRNA anti HBV PRE 1329-1349). (B) Detection of putative functional elements within the PRE by CDS-plotcon. PRE sequences from selected human HBV genotypes A-H were input into the CDS-plotcon programme. A high peak indicates conserved elements beyond that expected by coding. Four putative functional elements within HBV PRE were detected, at 1151-1310, 1390-1450, 1515-1610 and 1540-1684. The diagram was produced by CDS-plotcon online (C) Detection of conserved secondary RNA structures within the PRE. The same set of human HBV sequences was input into Alidot. The mountain plots indicate regions that contain conserved RNA secondary structures at 1151-1410, 1410-1433, 1440-1500 and 1530-1620. The dashed boxes indicate functional conserved elements that contain RNA secondary structures. The diagrams are to the same scale.

In this study, we were interested to identify siRNA targets within the HBV post-transcriptional regulatory element (HBV PRE), which is a *cis*-acting RNA element approximately 500 bases long found in all HBV transcripts. The PRE is a conserved RNA element [23] that has been reported to be involved in the regulation of HBV mRNAs including RNA splicing [24], RNA stability [25] and nuclear export [26-28]. In addition, its nuclear export is affected by myeloid differentiation primary response protein 88 (MyD88) [29]. Several regulatory elements have been identified within the PRE, including a human La binding site [30,31], stem-loop structures, HBV SLα and HBV SLβ [32], a *cis*-acting splicing regulatory element (SRE-1) [24], the binding sites (PRE III) of polypyrimidine tract binding protein (PTB) and glyceraldehydes-3-phosphate dehydrogenase (GAPDH) [33-36] (Figure 1A) and binding site of T-cell intracellular antigen 1 (TIA-1) [37], however, the core function of the PRE remains unclear. This study was therefore aimed to investigate potential siRNA target sites within the PRE as well as the core functional elements of the PRE.

## Materials and methods

### Bioinformatic analysis of functional elements

The functional elements within the PRE (nucleotides 1151-1684, of Accession number AM282986) were analyzed using results of CDS-Plotcon and Alidot programmes provided by the database HBVRegDB [23]. In brief, a set of 32 completed HBV genomes were analysed by the CDS-Plotcon programme [38] to specifically detect regulatory elements that are present within the coding sequence. They were also analysed using the Alidot programme [39] to determine conserved RNA secondary structures.

### Prediction of siRNA target sites

The siRNA target sites within the PRE sequence were predicted using siExplorer [40], siDirect [41], and siRNA target designer [42] programmes. Characteristics of desired siRNAs identified by Reynolds *et al *(2004) [22] were also taken into consideration. Nucleotide blast (blastn) was performed to check specificity of predicted siRNA target sites against human genomic and human transcript databases (Table 1 and additional file 1). The selected sequences were chosen to target PRE positions 1317-1337 and 1329-1349.

**Table 1:** Overlapping prediction of siRNA target sites found within HBV PRE by different bioinformatics tools.

Programme	Position	Sequence	GC (%)	Specificity (similarity %)	Position base preference				Reference
						
					A3*	T10*	Non G13*	A19*	
siExplorer	**1324-1343**	CAUCGGAACUGACAAUUCU	42.1	- 73% match ARP6 actin-related protein 6 homolog transcript^1^ - 79% match chromosome x genomiccontig^2^	T	T	C	T	[40]
	1640-1659	UGCCCAAGGUCUUACAUAA	42.1	- 74% match DIX domain containing 1 transcript^1^ - 100% match chromosome X genomic contig^2^	C	T	T	A	
	1641-1660	GCCCAAGGUCUUACAUAAG	47.3	- 73% match DIX domain containing 1 transcript^1^ - 95% match chromosome X genomic contig^2^	C	C	A	G	

siDirect	1344-1366	TCGTCCTCTCGCGGAAATATACA	52	- 66% match oligophrenin 1 (OPHN1) transcript^1^ - 71% chromosome 16 open reading frame 35^2^	G	C	G	A	[41]

siRNA target designer	1346-1364	GTCCTCTCGCGGAAATATA	47	- 73% match oligophrenin 1 (OPHN1) transcript^1^ - 74% match hypothetical protein LOC728975^2^	C	C	A	A	[42]
	**1321-1339**	GCTCATCGGAACTGACAAT	47	- 74% ma_1_tch tetraspanin 7 (TSPAN7) transcript^1^ - 84% match chromosome 13 contig^2^	T	A	T	T	

Reynolds et al (2004)	1317-1337*	AAAGCTCATCGGAACTGACAA	42	- 66% match tetraspanin 7 (TSPAN7) transcript^1^ - 81% match chromosome 2 genomic contig^2^	A	C	A	C	[22]
	**1318-1338**	AAGCTCATCGGAACTGACAAT	42	- 66% match tetraspanin 7 (TSPAN7) transcript^1^ - 95% match chromosome 17 genomic contig^2^	G	G	A	A	
	1329-1349*	AACTGACAATTCTGTCGTCCT	42	- 76% match zinc finger protein 559 (ZNF559) transcript^1^ - 76% match chromosome 19 genomic contig^2^	C	T	T	C	
	1336-1356	AATTCTGTCGTCCTCTCGCGG	52	- 71% match F-box and leucine-rich repeat protein 15 transcript^1^ - 81% match chromosome 20 genomic contig^2^	T	G	C	C	
	1357-1377	AAATATACATCGTTTCCATGG	33	- 62% match ADAM metallopeptidase domain 12 (ADAM12) transcript^1^ - 76% match chromosome 11 genomic contig^2^	A	T	T	T	
	1358-1378	AATATACATCGTTTCCATGGC	42	- 67% match bactericidal/permeability increasing protein-like 2 (BPIL2)^1^ - 76% match chromosome 11 genomic contig^2^	T	C	T	G	

Predicted siRNA target sites from siExplorer, siRNA target design and Reynolds *et al. *(2004) [22] were found to have overlapped regions. Bold indicates the position base preference. '*' indicates the selected siRNA target sites, '^1^' and '^2^' indicate the best matches from the blastn search against human transcript and genomic databases respectively. Details of the blastn matches are in additional file 1.

### Generation of luciferase reporter plasmids

An intronless reporter vector [pBasic (-IN)] was constructed by removing the chimeric intron of the pGL3MS2site/Basic reporter construct [43]. To construct a splicing luciferase reporter vector (pSpliceLuc), the parental vector, pGL3MS2site/Basic was modified to place the *luc+ *gene between the splicing donor (SD) and splicing acceptor (SA) sites. This modification involved two-step cloning. The first cloning involved amplification of the SD (forward: SV40+ 5'-ctgactaattttttttatttatgc-3', reverse: SDR_AflII 5'-gaattccttaagccttaaacctgtcttgtaacc-3') and then the amplification of the SA sequence (forward: SAF_XhoI 5'-gaattcctcgagagaccaatagaaactgggc-3', reverse: SAR_EcoRV 5'-gaattcgatatccctgtggagagaaaggcaaagtg-3').

### Amplification of the deletion series of the PRE

Three pairs of primers were specifically designed to amplify three sub-sections of the HBV PRE: (i) full length HBV PRE (forward: HBVPRE_1151F 5'-tctagagctagcttgct cggcaacggcctggtctgtg-3', reverse: HBVPRE_1684R 5'-gccggcctcgaggacattgctgaga gtccaagagtcc-3'); (ii) HBV PRE 1399-1684 (forward: HBVPRE_1399F 5'-tctagagc tagctggatccttcgcgggacgtcctttg-3', reverse: HBVPRE_1684R 5'-gccggcctcgagga cattgctgagagtccaagagtcc-3') and (iii) HBV PRE 1485-1584 (forward: HBV PRE_1485F 5'-tctagagctagctcgtccccttctccgtct-3', reverse: HBV PRE_1584R 5'-gccgg cctcgaggtgcacacggaccggcagat-3'). The Amplification was performed from a clone containing the complete HBV genome (a gift from M-H Lin, National Taiwan University) using Expand™High Fidelity DNA polymerase (Roche). Short fragments of the PRE 1292-1321 and HBV PRE 1411-1433 were created by annealing synthetic oligonucleotides: (i) forward: HBVSL_alpha oligoF 5'-ctagcgttttgctcgcagccggtctggggcaaagcc-3', reverse: HBVSL_alpha oligoR 5'-tcgaggctttgccccagaccggctgcgagcaaaacg-3'; and (ii) forward: HBVSL_beta oligoF 5'-ctagcgggacgtcctttgtttacgtcccc-3', reverse: HBVSL_beta oligoR 5'-tcgaggggacgtaaacaaaggacgtcccg-3'.

### Generation of siRNA expression plasmids

**Figure 2:**
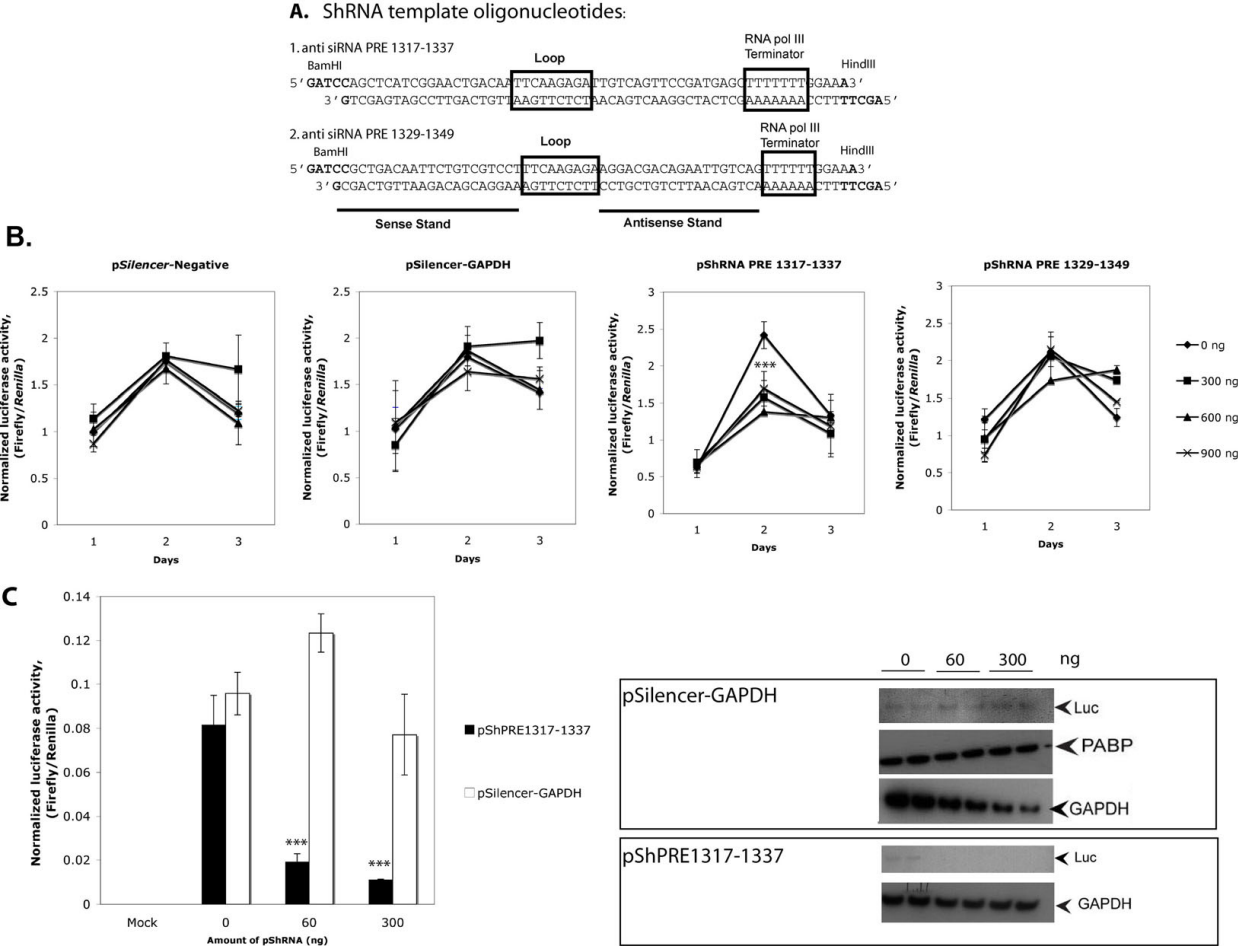
**The effect of siRNA expression plasmids on reporter activity**. (A) ShRNA template oligonucleotides of siRNA anti PRE 1317-1337 and PRE 1329-1349. (B) The effects of pShRNA plasmids on luciferase activity. The line graphs indicate normalized luciferase activities of transfected COS-7 cells with p*Silencer-*Negative, pSilencer-GAPDH, pShRNA PRE1317-1337 and pShRNA 1329-1349. COS-7 cells were transfected in triplicate with 95 ng of pLucSplice/fPRE, 5 ng of phRL-SV40 and various amount of pSiRNA expression plasmids as indicated, pUC18 was used to make up the total DNA to 1 μg. Cells were harvested at day 1, day 2 and day 3 post-transfection and analyzed for expression of luciferase protein. '***' indicates significant differences of normalized luciferase activities compared to 0 ng of siRNA expression plasmid with p < 0.001 (by t-test). (C) The effect of pShRNA PRE1317-1337 on the luciferase activity in HuH-7 cells. In this experiment, cells were transiently transfected in triplicate with 45 ng of the pSpliceLuc/fPRE, 5 ng of the phRL-SV40 and different amounts of the pShRNA constructs as indicated. pSilencer-Negative was used to make up total DNA plasmid to 350 ng. Cells were harvested and analyzed luciferase activity and western blot analysis after 48 h of incubation. Reporter expression levels are higher from these SV40 promoter containing constructs in COS- 7 cells (B) than in Huh-7 cells (C). The bar graph shows normalized luciferase activity of mean values of three independent experiments; error bars represent standard deviation. '***' indicates significant differences of normalized luciferase activities comparing to the control siRNA expression plasmid (pSilencer-GAPDH) with p < 0.001 (by t-test). Western blot analysis with primary antibodies: anti-GAPDH, anti-firefly luciferase protein (anti-Luc) and anti-Poly A binding protein (anti-PABP) and the horseradish peroxidase-conjugated secondary antibody.

Selected siRNA target sites at positions 1317-1337 and 1329-1349 were converted into shRNA template oligonucleotides by adding the loop sequence, the target site's antisense strand and a termination signal for the RNA pol III (Figure 2A). For cloning purposes, overhangs of half restriction enzyme sites for BamHI and HindIII were flanked at the 5'-end and the 3'-end of the template respectively. Sequences of the shRNA templates for targeting HBV PRE 1317-1337 and 1329-1349 were as follows: PRE1317 5'-gatccagctcatcggaactgacaattcaagagattgtcagttccgatgagctttttttggaaa-3'; 5AtPRE1317 5'-agcttttccaaaaaaagctcatcggaactgacaatctcttgaattgtcagttccgatgagctg-3'; PRE 1329 5'-gatccgctgacaattctgtcgtcctttcaagagaaggacgacagaattgtcagttttttggaaa-3'; AtPRE1329 5'-agcttttccaaaaaactgacaattctgtcgtccttctcttgaaaggacgacagaattgtcagcg-3'. Then, the two complementary hairpin siRNA oligonucleotides (each contains 1 μg/μL) were annealed together and ligated with the cut pSilencer 3.0-H1 promoter vector (Ambion).

### Western blot analysis

Cell lysates were separated on 4-12% Bis-Tris gel (NuPAGE^® ^Novex gel, InvitrogenTM Life Technologies) and electrophoretically transferred onto polyvinylidene difluoride (PVDF) membrane (Hybond-P, Amersham Pharmacia Biotech). Blots were blocked with 5% of skim milk in TBS-T buffer for 1 h and subsequently incubated at 4°C for overnight with appropriate diluted primary antibodies, anti-Luc (1:500, Roche), anti-GAPDH (1:2,500, Ambion) and anti-PABP (1:10,000, Abcam). Then blot was incubated with diluted horseradish peroxidase-conjugated secondary antibody, goat anti-mouse (1:10,000, BIO-RAD) at room temperature for 1 h. For chemiluminescent detection, the immuno-blot was applied with the ECL Plus reagents (Amersham Pharmacia Biotech) and exposed to X-ray films (HyperfilmTM, Amersham Bioscience) for 15 s - 10 min at room temperature. All exposed films were then processed and qualified by imaging densitometry (Molecular Analyst software).

### Mammalian tissue culture and transfection

HuH-7, HepG2 and COS-7 cells were cultured in 75 cm^3 ^sterile tissue culture flasks (Greiner Bio-One) at 37°C with 5% CO_2 _in DMEM supplemented (Invitrogen) with 10% heat inactivated FBS (10% v/v) (Invitrogen) and 1% L-glutamine (Invitrogen). Prior to transfection, cells were seeded on 24-well plates (Greiner Bio-One) with a cell density approximately 1X 10^4 ^cells/mL and incubated for 24 h. All transfection was performed using FuGENE6 (Roche). The ratio between FuGENE6 (μL) and DNA (μg) was 3:1. For deletion analysis, 195 ng of the deletion series of HBV PRE reporter plasmids were transiently co-transfected in quadruplicate with 5 ng of the internal control plasmid (phRL-SV40: Promega, a plasmid expressing humanized *Renilla *luciferase protein). pUC18 was used to top up the total amount of DNA if required. For evaluating effect of siRNA expression plasmids, cells were co-transfected in triplicate with 95 ng pSpliceLuc/fPRE or pBasic (-IN)/fPRE, 5 ng of phRL-SV40 and various amounts (0 ng, 300 ng, 600 ng and 900 ng) of the siRNA expression plasmids. For studying the siRNA effect of the pShRNA PRE 1317-1337 on the luciferase protein, cells were transiently co-transfected in triplicate with 45 ng of pSpliceLuc/fPRE or pBasic (-IN)/fPRE, 5 ng of the phRL-SV40 and either 60 ng or 300 ng of the pShRNA PRE 1317-1337 construct. The pSilencer-Negative was used to make up the total of plasmid DNA to 350 ng. The pSilencer-GAPDH was also included in the experiment as the positive control for the siRNA effect on the GAPDH protein.

### Luciferase activity assay

Forty-eight h post-transfection, cells were lysed with 100 μL of 1 × passive lysis buffer (Promega). Cell debris and nuclei were removed by centrifugation and the supernatant was collected. The luciferase activity assay was performed as described by Tanguay and Gallie (1996)

### Quantitative real time- PCR analysis of HBV cccDNA

A plasmid expressing covalently closed HBV genome (EMBL:AM282986) was constructed in pGEM-T Easy Vector (Promega, Madison, WI) through a T-A cloning strategy. Serial 10-fold dilutions of the cccDNA standard plasmid from 10 to 10^10 ^copies/μL were detected by real-time PCR assay and used to prepare the standard curve for quantitation of HBV cccDNA. The standard curve of HBV cccDNA was then constructed by plotting the logarithm of the initial plasmid concentration against the threshold cycle (Ct) obtained from each dilution. The standard plasmid DNA for quantitation was included in each run as an external standard. HBV cccDNA was amplified and quantified in real-time PCR assay using the primers and probe as described previously [44]. The forward primer was HBV_CCC_F1 (5'-actcttggactc cagcaatg-3'); the reverse primer was HBV_CCC_R1 (5'-ctttatacgggtcaatgtcca-3') and the cccDNA specific probe was FAM-ttcaagcctccaagctgtgccttg-BHQ1. The optimized real-time PCR reaction mixture comprised 1 μL of DNA template, 0.75 μM final concentration of each primer, 0.25 μL of the probe, 5 μL of 2 × Platinum qPCR Super Mix-UDG (Invitrogen, California, USA), additional 2.5 mM MgCl_2_, and nuclease-free water to a final volume of 10 μL. The real-time PCR assay was carried out in a Rotor Gene RG-3000 (Corbett Research, Australia) under the following conditions: initial denaturing step at 95°C for 10 min, followed by 45 cycles of 95°C for 15 s and 61.5°C for 1 min. Then the Rotor-Gene Software Version 6.0 (Corbett Research) was used for data acquisition and analysis of the HBV cccDNA level. The result was indicated in term of relative quantitation by comparative threshold (delta-delta Ct) method (2^- ΔΔCt^). The amount of target gene in the sample, normalized to an endogenous housekeeping gene (reference gene) and relative to the normalized calibrator, is then given by 2 ^- ΔΔCt^, where

ΔΔCt = ΔCt(sample) - ΔCt(calibrator)

ΔCt (sample) = Ct (target gene of sample) - Ct (reference gene of sample)

ΔCt (calibrator) = Ct (target gene of calibrator) - Ct (reference gene of calibrator)

Ratio (folds of difference) of sample: calibrator = 2 ^- ΔΔCt^

In this study, the reference gene was beta-globin, the target gene was the cccDNA of HBV co-transfected with siRNA, and the calibrator was cells transfected with only the HBV plasmid.

## Results and discussion

### The HBV PRE was predicted to contain multiple functional conserved elements

The HBV post-transcriptional regulatory element is highly conserved among the mammalian hepadnaviridae [23]. As the sequence of the PRE also encodes the P protein, the conservation may partly be due to constraints on the encoded protein. This study therefore analyzed functional core elements of the PRE using results generated by the CDS-plotcon programme, which scores conservation beyond what is required for coding [38] as well as using programmes for predicting conserved RNA secondary structure [39]. CDS-plotcon indicated four conserved elements within the functional PRE at nucleotide positions 1151-1310, 1390-1450, 1515-1610 and 1540-1684 (EMBL: AM282986, Figure 1B). Three of these potential conserved regulatory elements were predicted to form local RNA secondary structures by Alidot (Figure 1C). The results of both programmes therefore suggested three putative functional conserved elements at nucleotide positions 1151-1410, 1411-1433 and 1510-1620 (Dashed boxes, Figure 1).

Notably, the previously identified regulatory elements: the human La binding site [30], SRE-1 [24] and HBV SL alpha [32] are shown to be part of a large secondary RNA structure within the predicted functional element at the position 1151-1410 whereas the reported HBV SL beta [32] and the PRE III [34,35] are part of the identified functional elements at the position 1411-1433 and 1520-1620 respectively (Figure 1). In addition, known regulatory elements at the DNA level are also found to be part of the region identified as the functional elements (high mountain peak) generated by the CDS-plotcon such as the DNA enhancer 1 (nucleotide position 900-1310), the X promoter (nucleotide position 950-1310), the DNA primer binding DR1 (nucleotide position 1590-1600) and the DNA enhancer 2 (nucleotide position 1636-1744) [45,46].

Taken together, the results support that the PRE is an important regulatory region that contains multiple functional conserved elements.

### HBV PRE 1317-1377 is a novel siRNA target site

Although several sites in the HBV genome have previously been able to be targeted by siRNA, there is no report of targets within this region of PRE. To predict siRNA target sites, the sequence was analysed using three programmes, siExplorer [40], siDirect [41] and siRNA target designer [42]. In addition, the criteria of ideal siRNAs reported by Reynolds *et al *(2004) [22] were also taken into consideration. The predicted siRNA target sites were checked for specificity using nucleotide blast (blastn) against human genomic and transcript databases. Predicted siRNA target sequences that had more than 85% identity to human genomic DNA or transcripts were designated as non-specific siRNA targets and not used. As a result, an overlapping region of predicted siRNA target sites was detected by three different approaches (Table 1). Selected siRNA target sites were chosen to cover this region, at nucleotide position 1317-1337 and 1329-1349 (Figure 1A). These two predicted siRNA sites were found within the identified putative functional PRE element (nucleotide position 1151-1410) and they are part of a large predicted conserved RNA secondary structure (Figure 1). Selected siRNA target sites were converted into shRNA template oligonucleotides (Figure 2A) and ligated with the cut siRNA expression vector (pSilencer 3.0-H1, Ambion). The generated siRNA expression plasmids were designated as pShRNA PRE 1317-1337 and pShRNA PRE 1329-1349. Subsequently, various amounts (0 ng, 60ng, 300 ng, 600 ng and 900 ng) of the generated siRNA expression plasmids were transiently co-transfected in triplicate with 95 ng of luciferase reporter vector (pSpliceLuc/fPRE or pBasic (-IN)/fPRE) and 5 ng of *Renilla *expression plasmid (phRL-SV40) using FuGENE6. The experiment also included the positive control shRNA plasmid (pSilencer-GAPDH, Ambion), which targets the human GAPDH mRNA and the negative control plasmid (pSilencer-Negative, Ambion) a scambled sequence that is not found in the human genome. Cells were harvested at different time points (1 day, 2 days, 3 days post-transfection).

The result shows that the pShRNA PRE 1317-1337 could specifically and significantly reduce the level of luciferase activity at the day 2-time point (Figure 2B, p < 0.001) even with a low amount (60 ng) of the siRNA expression plasmid (Figure 2C). In contrast, the presence of pShRNA PRE 1329-1349 in different amounts showed no effect on luciferase expression at any time point (Figure 2B) although it was selected by similar criteria and position to the effective siRNA target site 1317-1337 (Table 1 and Figure 1). Therefore, the results suggest that specific properties of siRNA target sites are more significant than others for effective targeting. The level of pBasic (-IN)/fPRE was also significantly reduced by this siRNA (60 ng, by 43% and 300 ng, by 79%).

### Anti-HBV PRE 1317-1337 specifically reduced the level of cccDNA in transiently HBV infected cells

**Figure 3:**
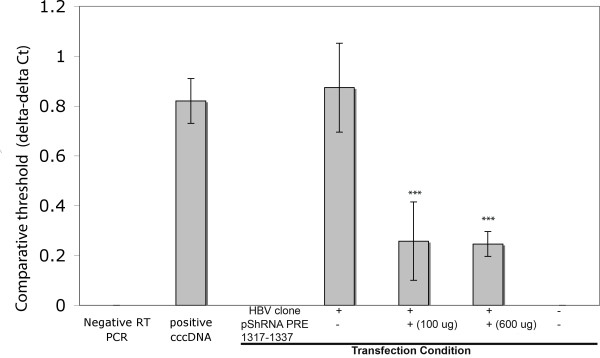
**The effect of pShRNA PRE 1317-1337 on the expression of cccDNA**. Bar graphs indicate mean values of thresholds of three independent experiments. In this study, HepG2 cells were transiently transfected in triplicate with 45 ng of the pSpliceLuc/fPRE, 5 ng of the phRL-SV40 and different amounts of the pShRNA constructs as indicated. pSilencer-Negative was used to make up total DNA plasmid to 350 ng. Cells were harvested and analyzed for luciferase activity and western blot analysis after 48 h of incubation. '***' indicates significant differences of comparative threshold comparing to the controls (positive cccDNA and cells without transfection of pShPRE 1317-1337) with p < 0.001 (by t-test).

This experiment was carried out to evaluate whether the anti-HBV PRE 1317-1377 could also inhibit HBV replication in infected cells. This was done by measuring the level of HBV covalently closed circular DNA (cccDNA) in HepG2 cells that were transiently co-transfected with 30 ng of a HBV clone that expresses HBV and with 0 ng, 100 ng or 600 ng of the pShRNA PRE 1317-1377. Forty eight h post-transfection, cells were harvested to analyze the level of cccDNA using quantitative real time PCR. The results indicated that the plasmid expressing siRNA anti-HBV PRE 1317-1377 significantly reduced the level of cccDNA in transiently infected cells (Figure 3, p < 0.001).

Previous reports showed that new formation of cccDNA in transfected cells was directly controlled by the expression of HBV transcripts [47,48]. As this siRNA target site (HBV PRE 1317-1337) is present in all HBV transcripts, it is possible that any or all HBV transcripts were reduced by the siRNA, resulting in the reduction of level of cccDNA.

### Sub-sections of the PRE have different effects on the reporter gene activity

**Figure 4:**
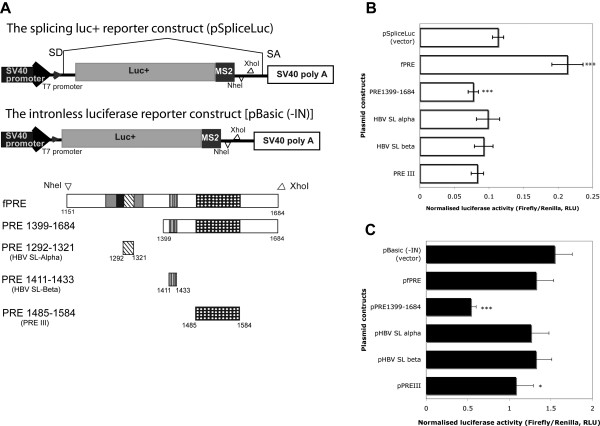
**The effects of sub-sections of the PRE on reporter activity**. (A) Schematic diagrams of Luc+ reporter constructs used in the study, the splicing Luc+ reporter constructs (pSpliceLuc) and the intronless luciferase reporter construct (pBasic (-IN)). Sub-sections of the PRE were indicated. Each HBV PRE sub-section was inserted into the pSpliceLuc vector at the NheI and XhoI sites. The numbering in the scheme corresponds to nucleotide number of HBV adw2 genotype A (EMBL:AM282986). (B) The ratios of test firefly luciferase and control *Renilla *luciferase proteins from the splicing luc+ reporter system (pSpliceLuc). (C) The ratios of firefly luciferase and *Renilla *luciferase proteins from the intronless luc+ reporter system pBasic (-IN)). The pSpliceLuc constructs used in B require PRE dependent prevention of splicing out the reporter from the intron, and thus have lower ratios than the intronless constructs used in C. In B and C, Cells were co-transfected with 195 ng of luciferase reporter plasmids, 5 ng of *Renilla *reporter. Forty-eight h post-transfection, HuH-7 cells were harvested and 10 μL of these cell lysates were assayed for expression of luciferase proteins using POLARstar OPTIMA (BMG Labtech). Each assay was repeated in triplicate. The mean values and standard deviations of three independent experiments of the normalized luciferase activities are shown. Error bars represent standard deviation. '*', '***' indicate significant differences of luciferase activities comparing to the control vector (pSpliceLuc) with p < 0.05 and p < 0.001 respectively.

To investigate the functional core elements of the PRE, a deletion series of the PRE was designed based on these predictions (CDS-plotcon and Alidot) and previous reports on PRE regulatory elements, HBV SL alpha (nucleotide position 1291-1321), PRE III (nucleotide position 1485-1584) (Figure 1). Each PRE subsection was then specifically generated by PCR and then inserted into two different luciferase (*luc+*) reporter constructs digested at NheI and XhoI sites: (i) the splicing *luc+ *reporter construct (pSpliceLuc), and (ii) the intronless luciferase reporter construct [pBasic (-IN)] (Figure 4A). Notably, the pSpliceLuc construct has the *luc+ *gene within an intron, thus it could be used to study whether the PRE could enhance the unspliced *luc+ *gene expression. On the other hand, the pBasic (-IN) reporter construct was designed to study functional nuclear export of PRE by imitating the natural context of the intronless HBV S transcript where PRE is located the 3' UTR of the gene. Subsequently, a deletion series of the PRE reporter plasmids were transiently co-transfected in quadruplicate with the phRL-SV40 vector in HuH-7 and COS-7 cells.

The full length (fPRE) significantly enhanced unspliced *luc+ *gene expression in both HuH-7 (Figure 4B, p < 0.001) and COS-7 cells (data not shown). This result suggests that the fPRE either inhibit splicing or enhance nuclear export, or both. Surprisingly, the PRE sub-section 1399-1684 significantly inhibited unspliced *luc+ *gene expression (p < 0.001) whereas PRE sub-section 1292-1321 (HBV SL alpha), HBV PRE 1411-1433 (HBV SL beta) and HBV PRE 1485-1584 (PRE III) did not individually enhance the expression of unspliced *luc+ *(Figure 4B). This result was consistent with previous published results, using the *cat *reporter system (pDM138), this construct has a design similar to the unspliced *luc+ *reporter construct used in this study. The previous report indicated that duplication of HBV SL beta and HBV SL alpha was required to enhance the level of CAT activity in the *cat *reporter system (pDM138) [26]. Indeed, six copies of HBV PRE III were reported to increase the CAT activity (pDM138 reporter system) in the same level as the full-length the PRE [49].

On the other hand, the results from the intronless *luc+ *construct showed that none of the PRE sub-sections including the fPRE were able to enhance the expression of the intronless *luc+ *gene. Interestingly, the PRE sub-section 1399-1684 also significantly inhibited the intronless *luc+ *gene whereas PRE alpha, PRE beta had no effect on the expression of intronless *luc+ *gene (Figure 4C). Previously using northern blot analysis and primer extension, the PRE has been shown to significantly increase cytoplasmic export of the HBV S RNA [27,50]. It has also been reported to function in the context of heterologous genes by enhancing expression of intronless transcripts of β-globin and c- myc [27,50-52]. In this study, the PRE failed to increase activity of the intronless Luc+ protein (Figure 4C). Therefore, experimental results from this study provide evidence suggesting that the ability of the PRE to enhance expression of intronless transcripts is not applicable to all intronless genes. It is possible that the PRE may not have an effect on the highly expressed gene *luc+ *whereas it did on poorly expressed reporters (e.g. *cat*). Therefore, the results of PRE deletion analysis from the intronless *luc+ *system might not be able to conclusively evaluate the identified functional elements. Subsequent studies should be conducted to test the function of these PRE elements in a natural context using pgRNA (C and P proteins) or surface (S) protein.

Interestingly, the PRE sub-section 1399-1684 significantly reduced luciferase activity (Figure 4B, p < 0.001). This result was also observed in the intronless *luc+ *reporter system (Figure 4C, p < 0.001). The result may suggest either that the PRE sub-section 1399-1684 contains a novel inhibitory element or that the PRE sub-section 1151-1398 is an important element for the function of the PRE. Taken together, the PRE appears to contain multiple weak regulatory elements, but some aspects regarding the function of the PRE are still unclear.

## Conclusion

In summary, we showed that the HBV PRE contains the effective siRNA target site (nucleotide position 1317-1337) that when targeted with shRNA could reduce the level of cccDNA in transiently transfected cells. However, more experiments are required to optimize the duration and efficiency of the siRNA effect. The computational and deletion analysis suggested that the HBV PRE is likely to contain several relatively weak regulatory elements that vary in conservation. These elements may have different functions during the HBV lifecycle.

## Competing interests

The authors declare that they have no competing interests.

## Authors' contributions

NP carried out: the bioinformatic analysis of functional elements and prediction of siRNA targets, plasmids' constructions, Western blot analysis, luciferase activity assay and drafted the manuscript. SP carried out the quantitative real-time-PCR analysis and participated in the manuscript. YP participated in the design of the study and analysis of the quantitative real-time-PCR study. CMB conceived of the study, and participated in its design and coordination. All authors read and approved the final manuscript.

## Supplementary Material

Additional file 1 **Blastn matches for potential siRNAs targeting the PRE region of HBV**. The file contains blastn results of potential siRNA target sites. The results indicate Score search, E-value and a list of matched sequences and sequence alignments. Click here for file
